# Draft Genome Sequence of *Brevibacillus panacihumi* Strain W25, a Halotolerant Hydrocarbon-Degrading Bacterium

**DOI:** 10.1128/genomeA.01215-13

**Published:** 2014-01-23

**Authors:** Xinxin Wang, Decai Jin, Lisha Zhou, Liang Wu, Wei An, Yu Chen, Lin Zhao

**Affiliations:** a School of Environmental Science and Engineering, Tianjin University, Tianjin, China; b China Offshore Environmental Service Co., Ltd., Tianjin, China; c College of Resources and Environment, Chinese Academy of Sciences, Beijing, China; d Shenzhen Key Laboratory of Environmental Microbial Genomics and Application, BGI, Shenzhen, China

## Abstract

*Brevibacillus panacihumi* strain W25 was isolated from hydrocarbon-contaminated saline soil. Here, we report the 5.5-Mb draft genome sequence of this strain, which may provide insights into the mechanism of microbial hydrocarbon degradation in saline environments.

## GENOME ANNOUNCEMENT

Microbial degradation plays a crucial role in the natural attenuation of hydrocarbon-contaminated saline environment, which has attracted increasing attention (1, 2). *Brevibacillus panacihumi* W25 was isolated from petroleum-contaminated saline soil in China, which can degrade crude oil in the presence of 40 g/liter NaCl. Although several strains belonging to the genus of *Brevibacillus* have been sequenced (3–9), the genome of *B. panacihumi* is still unavailable. Here, the draft genome sequence of *B. panacihumi* W25 is presented for the first time, helpful to gain a deeper understanding of the mechanisms of microbial hydrocarbon degradation in saline environments.

Genomic DNA was extracted from a 2-day culture growing in tryptic soy broth using a commercial DNA isolation kit, and was sequenced using the Illumina HiSeq 2000 platform (San Diego, CA). The shotgun sequencing yielded 10,458,934 read pairs (6,863,516 100-bp paired-end reads with an insert size of 500 bp and 3,595,418 100-bp mate-pair reads with an insert size of 6 kb). Filtered sequencing data were assembled using SOAPDeNovo v 2.04 (10) and were scaffolded and gap filled with SSPACE v 2.0 (11) and GapFiller v 1.10 (12). Final assembly consisted of 18 contigs with an *N*_50_ length of 1,095,539 bp, which were assembled into 11 scaffolds with an *N*_50_ length of 1,760,181 bp. The sequences were annotated using the NCBI Prokaryotic Genome Automatic Annotation Pipeline (PGAAP) (https://www.ncbi.nlm.nih.gov/genome/annotation_prok/).

The genome consists of 5.5 Mb with a G+C content of 50.1%. A total of 5,294 coding sequences (CDS), 115 pseudogenes, 135 tRNA genes, 1 noncoding RNA (ncRNA), and 11 rRNA operons were identified. One clustered regularly interspaced short palindromic repeat (CRISPR) element with 75 spacers was detected using CRISPR Finder (13). Eighty-seven tandem repeats was detected using Tandem Repeats Finder v 4.07 (14). One intact prophage sequence was identified using PHAST (15). The IS3 family dominates the insertion sequence (IS) elements, as revealed by ISFinder (16). Average nucleotide identity (ANI) analysis (17) revealed that *B. panacihumi* W25 is phylogenetically related to *B. agri* BAB-2500 (74.2%) (6), *B. borstelensis* AK1 (71.6%), *B. brevis* FJAT-0809-GLX (72.2%) (3), *B. brevis* NBRC100599 (72.5%), *B. brevis* X23 (72.3%) (4), *B. laterosporus* DSM 25 (66.4%), *B. laterosporus* GI 9 (67.4%) (9), *B. laterosporus* LMG 15441 (67.6%) (5), *B. laterosporus* PE36 (67.6%), *B. massiliensis* phR (69.2%) (8), *Brevibacillus* sp. BC25 (72.2%), *Brevibacillus* sp. CF112 (74.3%) and *B. thermoruber* 423 (72.6%) (7).

Seven genes were identified as involved in hydrocarbon degradation, including 1 alkanal monooxygenase gene and 6 ring-cleavage dioxygenase genes. Moreover, 7 genes were identified as involved in compatible solute synthesis and uptake, including 1 ectoine synthase gene, 1 betaine-aldehyde dehydrogenase gene and 5 glycine/betaine ABC transport genes, which may enhance the tolerance to NaCl stress. Information about the genome sequence of *B. panacihumi* W25 will improve our understanding of microbial hydrocarbon degradation in saline environment.

### Nucleotide sequence accession number.

The draft genome sequence of *B. panacihumi* W25 has been deposited in GenBank under the accession number AYJU00000000. The version described in this paper is the first version.

## References

[1] Mahmoudi N Porter TM Zimmerman AR Fulthorpe RR Kasozi GN Silliman BR Slater GF 2013 Rapid degradation of Deepwater Horizon spilled oil by indigenous microbial communities in Louisiana salt marsh sediments. Environ. Sci. Technol. 47:13303–13312. 10.1021/es4036072 24219093

[2] Wang X Han Z Bai Z Tang J Ma A He J Zhuang G 2011 Archaeal community structure along a gradient of petroleum contamination in saline-alkali soil. J. Environ. Sci. 23:1858–1864. 10.1016/S1001-0742(10)60640-7 22432311

[3] Che J Liu B Lin Y Tang W Tang J 2013 Draft genome sequence of biocontrol bacterium *Brevibacillus brevis* strain FJAT-0809-GLX. Genome Announc. 1(2):e00160-13. 10.1128/genomeA.00160-13 23618715 PMC3636543

[4] Chen W Wang Y Li D Li L Xiao Q Zhou Q 2012 Draft genome sequence of *Brevibacillus brevis* strain X23, a biocontrol agent against bacterial wilt. J. Bacteriol. 194:6634–6635. 10.1128/JB.01312-12 23144389 PMC3497470

[5] Djukic M Poehlein A Thürmer A Daniel R 2011 Genome sequence of *Brevibacillus laterosporus* LMG 15441, a pathogen of invertebrates. J. Bacteriol. 193:5535–5536. 10.1128/JB.05696-11 21914864 PMC3187376

[6] Joshi MN Sharma A Pandit AS Pandya RV Saxena AK Bagatharia SB 2013 Draft genome sequence of *Brevibacillus* sp. strain BAB-2500, a strain that might play an important role in agriculture. Genome Announc. 1(1):e00021-13. 10.1128/genomeA.00021-13 23472223 PMC3587923

[7] Yasar Yildiz S Kambourova M Arga KY Toksoy Oner E 2013 Draft genome sequence of exopolysaccharide-producing thermophilic bacterium *Brevibacillus thermoruber* strain 423. Genome Announc. 1(5):e00774-13. 10.1128/genomeA.00774-13 24072869 PMC3784789

[8] Hugon P Mishra AK Nguyen T-T Raoult D Fournier P-E 2013 Non-contiguous finished genome sequence and description of *Brevibacillus massiliensis* sp. nov. Stand. Genomics Sci. 8:1–14. 10.4056/sigs.3466975 PMC3739172 23961307

[9] Sharma V Singh PK Midha S Ranjan M Korpole S Patil PB 2012 Genome sequence of *Brevibacillus laterosporus* strain GI-9. J. Bacteriol. 194:1279–1279. 10.1128/JB.06659-11 22328768 PMC3294783

[10] Luo R Liu B Xie Y Li Z Huang W Yuan J He G Chen Y Pan Q Liu Y Tang J Wu G Zhang H Shi Y Liu Y Yu C Wang B Lu Y Han C Cheung DW Yiu SM Peng S Xiaoqian Z Liu G Liao X Li Y Yang H Wang J Lam TW Wang J 2012 SOAPdenovo2: an empirically improved memory-efficient short-read de novo assembler. Gigascience 1:18. 10.1186/2047-217X-1-18 23587118 PMC3626529

[11] Boetzer M Henkel CV Jansen HJ Butler D Pirovano W 2011 Scaffolding pre-assembled contigs using SSPACE. Bioinformatics 27:578–579. 10.1093/bioinformatics/btq683 21149342

[12] Boetzer M Pirovano W 2012 Toward almost closed genomes with GapFiller. Genome Biol. 13:R56. 10.1186/gb-2012-13-6-r56 22731987 PMC3446322

[13] Grissa I Vergnaud G Pourcel C 2007 CRISPRFinder: a web tool to identify clustered regularly interspaced short palindromic repeats. Nucleic Acids Res. 35:W52–W57. 10.1093/nar/gkm360 17537822 PMC1933234

[14] Benson G 1999 Tandem repeats finder: a program to analyze DNA sequences. Nucleic Acids Res. 27:573–580. 10.1093/nar/27.2.573 9862982 PMC148217

[15] Zhou Y Liang Y Lynch KH Dennis JJ Wishart DS 2011 PHAST: a fast phage search tool. Nucleic Acids Res. 39:W347–W352. 10.1093/nar/gkr485 21672955 PMC3125810

[16] Siguier P Perochon J Lestrade L Mahillon J Chandler M 2006 ISfinder: the reference centre for bacterial insertion sequences. Nucleic Acids Res. 34:D32–D36. 10.1093/nar/gkj014 16381877 PMC1347377

[17] Richter M Rosselló-Móra R 2009 Shifting the genomic gold standard for the prokaryotic species definition. Proc. Natl. Acad. Sci. U. S. A. 106:19126–19131. 10.1073/pnas.0906412106 19855009 PMC2776425

